# Assessment of accuracy and repeatability of quantitative parameter mapping in MRI

**DOI:** 10.1007/s12194-024-00836-4

**Published:** 2024-08-28

**Authors:** Yuya Hirano, Kinya Ishizaka, Hiroyuki Sugimori, Yo Taniguchi, Tomoki Amemiya, Yoshitaka Bito, Kohsuke Kudo

**Affiliations:** 1 Department of Radiological Technology, Hokkaido University Hospital, N15, W7, Kita-Ku, Sapporo, 060-8638 Japan; 2 Faculty of Health Sciences, Hokkaido University, Sapporo, 060-0812 Japan; 3 Clinical AI Human Resources Development Program, Faculty of Medicine, Hokkaido University, Sapporo, Hokkaido 060-8648 Japan; 4 Medical Systems Research & Development Center, FUJIFILM Corporation, Tokyo, Japan; 5 FUJIFILM Healthcare Corporation, Tokyo, Japan; 6 Department of Diagnostic Imaging, Graduate School of Medicine, Hokkaido University, N15, W7, Kita-Ku, Sapporo, 060-8638 Japan; 7 Global Center for Biomedical Science and Engineering, Faculty of Medicine, Hokkaido University, N15, W7, Kita-Ku, Sapporo, 060-8638 Japan

**Keywords:** Magnetic resonance imaging, Multi-parameter mapping, Relaxation time, Bloch simulation

## Abstract

We aimed to evaluate the accuracy and repeatability of the *T*1, *T*2*, and proton density (PD) values obtained by quantitative parameter mapping (QPM) using the ISMRM/NIST MRI system phantom and compared them with computer simulations. We compared the relaxation times and PD obtained through QPM with the reference values of the ISMRM/NIST MRI system phantom and conventional methods. Furthermore, we evaluated the presence or absence of influences other than noise in *T*1 and *T*2* values obtained by QPM by comparing the obtained coefficient of variation (CV) with simulation results. The *T*1, *T*2*, and PD values by QPM showed a strong correlation with the measured values and the referenced values. The simulated CVs of QPM calculated for each sphere showed similar trends to those of the actual scans.

## Introduction

Qualitative and subjective evaluations using image contrast, such as *T*1 or *T*2 weighted image (WI)s of magnetic resonance imaging (MRI), have traditionally been the primary methods for clinical image interpretation and diagnosis. Meanwhile, quantitative values, such as material-specific *T*1, *T*2, and proton density (PD) values, have become available for quantitative and objective evaluations. For example, *T*1 mapping using the modified Look–Locker sequence (MOLLI) has been widely used in cardiac regions to characterize myocardial infarction and cardiac amyloidosis [1]. Similarly, *T*2 mapping has also been employed to evaluate the knee cartilage [2]. Clinical applications of PD mapping comprise the investigation of patients with hepatic encephalopathy [3]. In these methods, one type of quantitative image is acquired using a single scanning sequence.

Recently, more advanced techniques capable of simultaneously acquiring several quantitative images through a single scanning sequence have been developed, such as synthetic MRI [4] with quantification of relaxation times and PD by multi-echo acquisition of saturation recovery with TSE readout (QRAPMASTER) and MR fingerprinting (MRF) [5]. These quantitative techniques initially focused on 2D scans; however, 3D scans have also been proposed in recent years [6]. Quantitative parameter mapping (QPM), a 3D quantitative technique, enables 3D scans based on partially RF-spoiled gradient echo (pRSGE) [7]. It is a quantitative imaging technique that enables the simultaneous estimation of *T*1 and *T*2* relaxation times, proton density, B1, and quantitative susceptibility mapping (QSM) by fitting an intensity function obtained through numerical simulation [7].

Because quantitative evaluations are expected to be used for various diagnostic purposes, such as follow-up [8] and tissue classification [9], the accuracy and repeatability of these scan sequences are very important. Superimposition error can vary greatly depending on the *T*1 and *T*2 values in a complex measurement technique, such as QPM. Therefore, it is important to evaluate it based on the actual scan regions and scan parameters. In a study by Taniguchi et al., the accuracy and repeatability of QPM were evaluated through short-term (10 measurements) continuous imaging of phantoms with relaxation times close to the optimization targets of QPM, namely *T*1 and *T*2 [10]. Although QPM was shown to have short-term repeatability of targeted *T*1 and *T*2*, it has not been shown to have long-term repeatability of a wide range of *T*1 and *T*2* in addition to PD. Only *T*1 and *T*2* values were assessed for accuracy, not PD values.

In this study, we aimed to evaluate the accuracy of the quantitative *T*1, *T*2*, and PD values obtained by QPM using the ISMRM/NIST MRI system phantom, which possesses a wide range of *T*1, *T*2, and PD values. Coefficients of variation (CV) were used to evaluate repeatability over a 15-day period.

## Theory

QPM is a method that simultaneously obtains *T*1, *T*2*, PD, and QSM maps using a 3D pRSGE. Several quantitative values are estimated by taking multiple image scans while varying a set of four scan parameters (flip angle [FA], RF-pulse phase increment [*θ*], TR, and TE) and finding a least-squares fit for the intensity function, which expresses the relationship between scan parameters and intensity values. [10] Fitting formula (1) is as follows:

1 $$\begin{gathered} x^{2} = \mathop \sum \limits_{\theta } \mathop \sum \limits_{{{\text{TR}}}} \mathop \sum \limits_{{{\text{FA}}}} \left\{ {{\text{I}}\left( {{\text{TR}},{\text{FA}},\theta ,{\text{TE}}} \right) - {\text{f}}\left( {T{1},T2{*},{\text{PD}},{\text{B}}1,{\text{FA}},{\text{TR}},\theta ,{\text{TE}}} \right)} \right\}^{2} = {\text{min}}. \hfill \\ \;\;\;\;\;\;\;\;\;\;\;0.0{5}\, < \,T{1}\, < \,{5}.{6}, \, 0.0{1}\, < \,T{2}\, < \,{2}.{8}, \, 0.{3}\, < \,{\text{B}}\_{1}\, < \,{1}.{5},\;0\, < \,{\text{PD}}, \hfill \\ \end{gathered}$$

where *θ* is phase cycling of RF, TR is the repetition time, FA is the flip angle, I(FA, TE, *θ*, TE) is the intensity of acquired QPM from dataset images, and *f* is the intensity function. The intensity function *f* expresses the luminance value using the subject's tissue parameters and imaging parameters as variables. The intensity function is created using Bloch simulation because it is analytically very complex. The Bloch simulation simulates magnetic resonance signal by taking as input the subject model (PD, *T*1, *T*2, distributions), imaging sequence, and imaging conditions, and solves the Bloch equations. The parameters *T*1, *T*2*, and PD are estimated by fitting the obtained data with this formula. A susceptibility map is estimated using a conventional method [11] from the multi-echo image obtained by the QPM scan. A QPM scan parameter set is optimized by simulating the combination of FA, TR, and *θ* using the error propagation method to minimize the fitting error of the *T*1 and *T*2* values of the target scan tissue. The number of scan parameter sets is specified during optimization, taking into account the imaging time and other factors.

## Materials and methods

### Subject

An International Society of Magnetic Resonance in Medicine/National Institute of Standards and Technology (ISMRM/NIST) system phantom (High Precision Devices, Inc., Boulder, CO, USA) was used to evaluate the accuracy and repeatability of the QPM. The details of the ISMRM/NIST MRI system phantom configuration can be confirmed at the following site (https://qmri.com/qmri-solutions/t1-t2-pd-imaging-phantom/). The *T*1, *T*2, and PD values for each array are listed in Table 1.

**Table 1:** *T*1 and *T*2 values for the *T*1 array and *T*2 array of the ISMRM/NIST MRI system

	*T*1 array			*T*2 array			PD array
	*T*1 value (ms)	*T*2 value (ms)		*T*1 value (ms)	*T*2 value (ms)		Concentration (% water)
*T*1-1	1838	1354	*T*2-1	2756	645.8	PD-1	5
*T*1-2	1398	1035	*T*2-2	2281	423.6	PD-2	10
*T*1-3	998.3	728.3	*T*2-3	1961	286	PD-3	15
*T*1-4	725.8	524.4	*T*2-4	1552	184.8	PD-4	20
*T*1-5	509.1	368.6	*T*2-5	1341	134.1	PD-5	25
*T*1-6	367	266.7	*T*2-6	1017	94.4	PD-6	30
*T*1-7	258.7	189.3	*T*2-7	782.1	62.51	PD-7	35
*T*1-8	184.7	134.1	*T*2-8	589.7	44.98	PD-8	40
*T*1-9	130.8	93.8	*T*2-9	443.8	30.95	PD-9	50
*T*1-10	90.9	65.7	*T*2-10	229.8	20.1	PD-10	60
*T*1-11	64.2	46.8	*T*2-11	237.8	15.4	PD-11	70
*T*1-12	46.28	33.11	*T*2-12	170.5	10.85	PD-12	80
*T*1-13	32.65	23.69	*T*2-13	121.8	7.59	PD-13	90
*T*1-14	22.95	16.73	*T*2-14	86.9	5.35	PD-14	100

### MRI scan

**Fig. 1:**
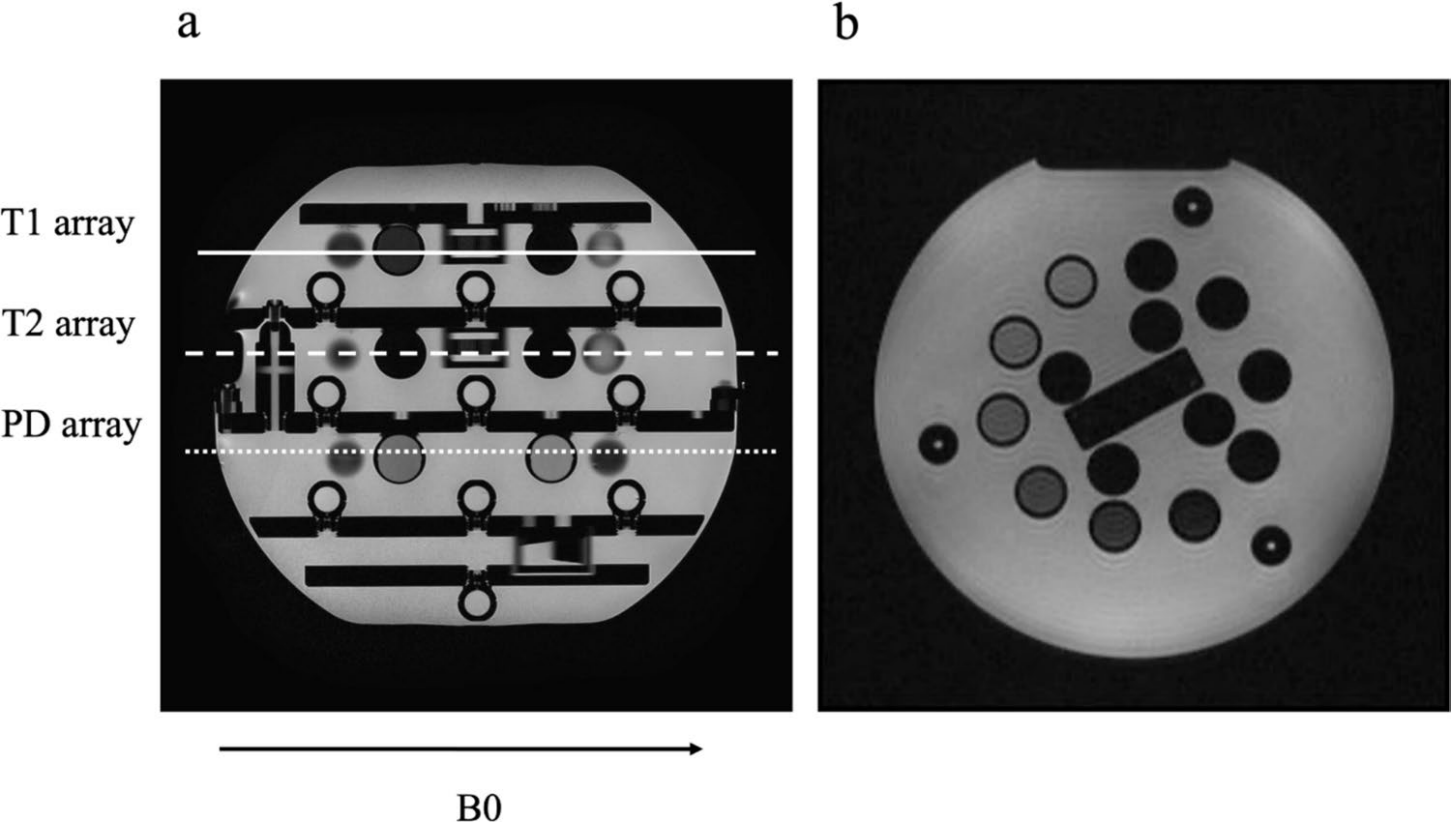
**a** sagittal T2WI of ISMRM/NIST MRI system phantom. The *T*1 array (solid line), *T*2 array (dashed line), and PD array (dotted line) are placed so that they are vertical to B0 (arrow). **b** Axial T2WI along with the *T*2 array

The ISMRM/NIST MRI system phantom was scanned using a 3 T MR scanner (Fujifilm, Ltd., Tokyo, Japan) equipped with a 15-channel head coil. The air-conditioning temperature of the MR scanner room was fixed at 22 °C, and a bandgap temperature sensor (standard accuracy 0.4 °C, resolution 0.01 °C) was placed 130 cm behind the gantry and 100 cm above the floor. The temperature measured hourly during the scans was 20.8 ± 0.5 °C. The ISMRM/NIST MRI system phantom was placed in the head coil such that the five plate surfaces were vertical to B0. The phantom was placed at the center of the gantry for 30 min before the scans. Axial images were scanned such that 14 spheres were visible in one image (Fig. 1). QPM, 2D inversion recovery spin echo (IR-SE), and 2D multi-echo RSGE were scanned from April 9, 2021, to April 24, 2021. The QPM, IR-SE, and RSGE were continuously scanned as a single set. Fifteen sets were obtained, with intervals larger than 6 h.

The optimization of QPM scan parameter sets (TR, TE, FA, and *σ*) were determined using the *T*1 and *T*2* values of the human brain tissues (gray matter (GM), white matter (WM), fat, and cerebrospinal fluid (CSF)) in Table 2 as target tissues [10]. Table 3 shows the scan parameter set of QPM used in this study. Other scan parameters were set as follows: FOV, 224 × 224mm^2^; slice thickness, 1.2 mm; number of slice, 187; acceleration factor, 1.2 × 1.2; matrix, 188 × 188; voxel size, 1.19 × 1.19 × 1.20; bandwidth, 90 kHz; number of signals averaged, 1. In this study, 11 different 3D images were acquired using these multiple imaging parameters in five scans, and the total acquisition time was 41 min and 26 s.

**Table 2:** Estimated *T*1 and *T*2* values for determining scan parameters in the brain QPM

	*T*1 (ms)	*T*2* (ms)
GM	1500	100
WM	850	75
Fat	300	70
CSF	3000	1200

**Table 3:** The QPM scan parameters (TE, FA, TR, and *θ*) calculated assuming that the brain is the imaging target

Scan no	FA (deg)	TR (ms)	*θ* (deg)	TE (ms)
1	10	40	20	4.6/11.5/18.4/25.3/32.2
2	25	20	22	4.6/9.2/13.8
3	35	40	2	4.6
4	40	20	5	4.6
5	40	10	8	4.6

IR-SE for *T*1 value measurement and RSGE for *T*2* value measurement were performed using the imaging parameters in Table 4.

**Table 4:** Scan parameters for IR-SE and RSGE

	IR-SE		RSGE
TR (ms)	10,000		500
TE (ms)	15		4.6, 14.6, 24.6, 34.6, 44.6, 54.6, 64.6
TI (ms)	50, 100, 200, 400, 800, 1600, 3200		–
Matrix size	188 × 188		
FOV (mm^2^)	224 × 224		
Slice thickness (mm)	1.2		
Scan time	3 h 40 min 37 s		1 min 34 s

### Data analysis

*T*1, *T*2*, and PD maps of QPM were created by performing the fitting process of Eq. (1) for each voxel using the acquired 11 3D images. Because the fitting process is limited to the calculation range of the *T*1 and *T*2* values (*T*1 value: 50–5600 ms, *T*2 value: 10–2800 ms), spheres No. 12–14 of *T*1 and *T*2 were excluded from the evaluation for accurate analysis. *T*1 and *T*2* values were calculated by IR-SE and RSGE, respectively, with the following procedure using MATLAB 2014b (MathWorks, Natick, MA, USA). The signal intensities *M* measured by each TI or TE image were substituted into Eqs. (2) and (3), respectively, and the *T*1 and *T*2* values were calculated by performing the nonlinear approximation process of the Levenberg–Marquardt method.

2 $$M = M0 (1-2 \left(\text{exp }\left(- T\text{I}/T1\right)\right),$$

3 $$M = M0\text{ exp }\left(-{\text{TE}/{T2}^{*}}\right),$$

where *M*0 is the equilibrium magnetization and *M* is any remnant magnetization that still presents at time *t* from any previous manipulations. Because *T*2* value analysis using targets with short *T*2 values might result in errors in *T*2* estimates due to noise effects caused by the inclusion of long TE signals, *T*2*map estimation was performed on two echoes at 4.6 ms and 14.6 ms for spheres with short *T*2 values (*T*2–11,10) and all seven echoes for other spheres to minimize the effect of noise.

### Simulations

We calculated the coefficients of variation (CV) from computer simulations that considered only normal distribution noise and compared it with the CV from *T*1 and *T*2* values obtained by QPM measurements to assess whether there are influences other than noise in the QPM measurements. Possible influences other than noise in the actual measurements include B1 intensity, spoilage accuracy, and liquid motion. The CV of QPM, IR-SE, and RSGE were calculated using computer simulations. The calculation algorithm used a Monte Carlo simulation to consider the noise effects. In this simulation, *T*2* is denoted as *T*2 because the effect of B0 inhomogeneities owing to differences in magnetic susceptibility is not included. All simulations were performed using Mathematica software (Wolfram Research, Champaign, IL, USA).

#### QPM simulations

The CV of the 11 spheres in each of the *T*1 and *T*2 arrays were calculated using the following steps. Step 1: Substitute the *T*1 and *T*2 values and the 11 scan parameter sets of QPM into the QPM intensity function *f*(*T*1, *T*2, PD, B1, FA, TR, θ, TE). Here, the PD and B1 were set to 1. In this context, PD is set to 1 as the proportional coefficient to intensity values, and B1 is set to a uniform value of 1 as the spatial distribution coefficient of FA. This yielded 11 spheres × 11 parameter intensities for each of the *T*1 and *T*2 arrays. Step 2: Add 900 Gaussian noise to each of these 11 × 11 intensities and calculate 11 × 11 × 900 intensities with noise added. Step 3: Estimate *T*1 and *T*2 values for these intensities using QPM and obtain 11 spheres × 2 arrays × 900 pairs of (*T*1, *T*2). Step 4: Calculate CV from the estimated T1 and T2 values for each sphere.

#### IR-SE simulations

The *T*1 and *T*2 values of the 11 spheres in the *T*1 array and the seven scan parameter sets of the IR-SE sequence with different TI were substituted into the *T*1 relaxation equation shown below to obtain 11 spheres × 7 parameter intensities.

4 $$I1=a1 {\text{Exp}}\left(-\text{TE}/T2\right) \left|1-2 B1 {\text{Exp}}\left(-\text{TI}/T1\right)\right|,$$

where $I1$ is the intensity, $a1$ is the proportionality coefficient, and $\text{TI}$ is the inversion time. Nine-hundred Gaussian noises were added to these intensities to obtain 11 × 7 × 900 intensities with added noise. For each intensity, a least-squares fit of Eq. 2 was found to obtain the *T*1 value, and the CV was calculated from the 900 *T*1 values for each of the 11 spheres.

#### RSGE simulations

The *T*1 and *T*2 values of the 11 spheres in the *T*2 array and the seven scan parameter sets of the RSGE sequence with different TE were substituted into the *T*2 relaxation equation shown below to obtain 11 spheres × 7 parameter intensities.

5 $$I2=a2 {\text{Exp}}\left(-\text{TE}/T2\right),$$

where $I2$ and $a2$ are intensity and the proportionality coefficient, respectively. Nine hundred Gaussian noises were added to these intensities to obtain 11 × 7 × 900 intensities with added noise. For each intensity, a least-squares fit of Eq. 3 was used to obtain a *T*2 value, and the CV was calculated from the 900 *T*2 values for each of the 11 spheres. Because the signal intensity in the *T*2-10 and *T*2-11 spheres with short T2 values decayed in a short time, the *T*2 values were calculated from least-squares fitting using only the two scan parameters with short TE, as in the actual scans.

### Evaluation

In this study, T1 maps of the QPM and IR-SE were used for *T*1 value evaluation, and *T*2* maps of the QPM and RSGE were used for *T*2* value evaluation. The PD maps obtained from QPM were used to evaluate the PD values. In addition to directly evaluating PD values, normalized PD with PD-14 (water, 100%), which means the percentage unit (pu) [12], was also evaluated because PD measured by QPM is a relative value. The measured value obtained from the PD map was defined as the unnormalized PD, and the normalized value from PD-14 was defined as the normalized PD. Region of interest (ROI) measurements for *T*1, *T*2*, and PD maps of the QPM were performed using ImageJ (National Institutes of Health, software version:1.52d). Rectangular ROIs (100 mm^2^) were placed at the center of the spheres by one of the authors (Y. H.). IR-SE and RSGE were measured by manually placing the ROIs in the same manner.

The mean and SD of *T*1 values were calculated and compared among the *T*1 maps of QPM, IR-SE, and the *T*1 reference values of the ISMRM/NIST MRI system phantom, and *T*2* values were calculated and compared among QPM, RSGE, and *T*2 reference values using intraclass correlation coefficients (ICC) and Bland–Altman plots. Additionally, ICC and Bland–Altman plots were generated using the normalized PD values obtained from QPM and the PD reference values of the ISMRM/NIST system phantom. JMP pro14 (SAS Institute Inc, Cary, NC, USA) and IBM SPSS Statistics version 21 (IMB, Armonk, NY, USA) were used for the statistical analyses. The repeatability of QPM was characterized by the CV and compared with the coefficient of variation of IR-SE and RSGE. CV was defined as the ratio between the standard deviation and mean *T*1, *T*2*, and PD values of 15 measurements. In addition, the measured CVs and computer simulations were compared to evaluate factors other than normally distributed noise that affect the quantitative values of QPM.

## Results

**Fig. 2:**
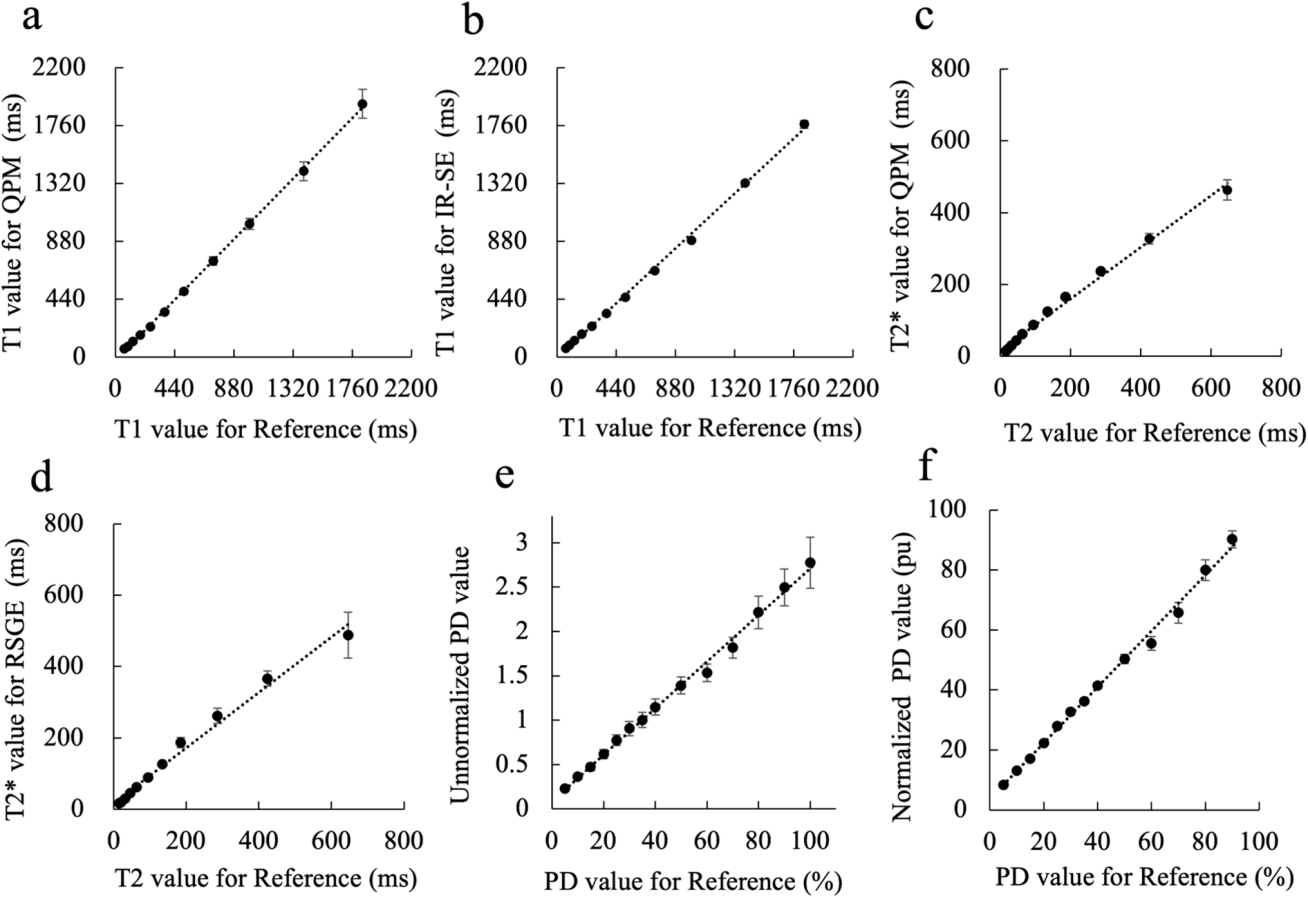
The correlation plots show the average *T*1 values of QPM (**a**) and IR-SE (**b**) for each *T*1 array compared with the reference *T*1 values. The ICC (2, 1) for the *T*1 value of QPM (**a**) and IR-SE (**b**) compared to the reference *T*1 value is 0.999 (95% CI 0.995–1.000) and 0.996 (95% CI 0.931–0.999), respectively. The slopes of the linear regression were QPM (1.047) and IR-SE (0.951). Moreover, we show the average *T*2* (or *T*2) values of QPM (**c**) and RSGE (**d**) for each T2 array compared with the reference *T*2 values. The ICC (2, 1) for the *T*2* value of QPM (**c**) and RSGE (**d**) compared to the reference *T*2 value is 0.934 (95% CI 0.750–0.982) and 0.960 (95% CI 0.858–0.989), respectively. The slopes of the linear regression were QPM (0.720) and RSGE (0.777). The correlation plots show the unnormalized PD values (**e**) and normalized PD values (**f**) for each PD array compared with the reference PD values. The ICC (2, 1) for the normalized PD values compared with the reference PD values was 0.995 (95% CI 0.985–0.998). The slope of the linear regression between the unnormalized PD value and the reference PD value was 0.996, while the normalized PD value was 0.995

Figure 2 shows the relationship between the *T*1, *T*2*, unnormalized PD, and normalized PD values obtained by QPM/IR-SE/RSGE and the reference values of the ISMRM/NIST MRI system phantom. The correlations to reference values were excellent for both QPM and IR-SE, as the ICC (2, 1) for the *T*1 values of QPM and IR-SE compared to the reference *T*1 value was 0.999 (95% CI 0.995–1.000) and 0.996 (95% CI 0.931–0.999), respectively. The slopes of the linear regression were near 1.0 both for QPM (1.047) and IR-SE (0.951). In contrast, the *T*2* values were moderate for *T*2* for both QPM and RSGE, as the slopes of the linear regression were 0.720 and 0.777 for QPM and RSGE, respectively. The ICC (2, 1) for the *T*2* value of QPM and RSGE compared with the reference *T*2 value was 0.934 (95% CI 0.750–0.982) and 0.960 (95% CI 0.858–0.989), respectively. The slope of the linear regression between the unnormalized PD value measured by QPM and the reference PD value was 0.996, while the normalized PD value was 0.995. The ICC (2, 1) for the normalized PD values obtained from QPM compared with the reference PD values was 0.995 (95% CI 0.985–0.998).

**Fig. 3:**
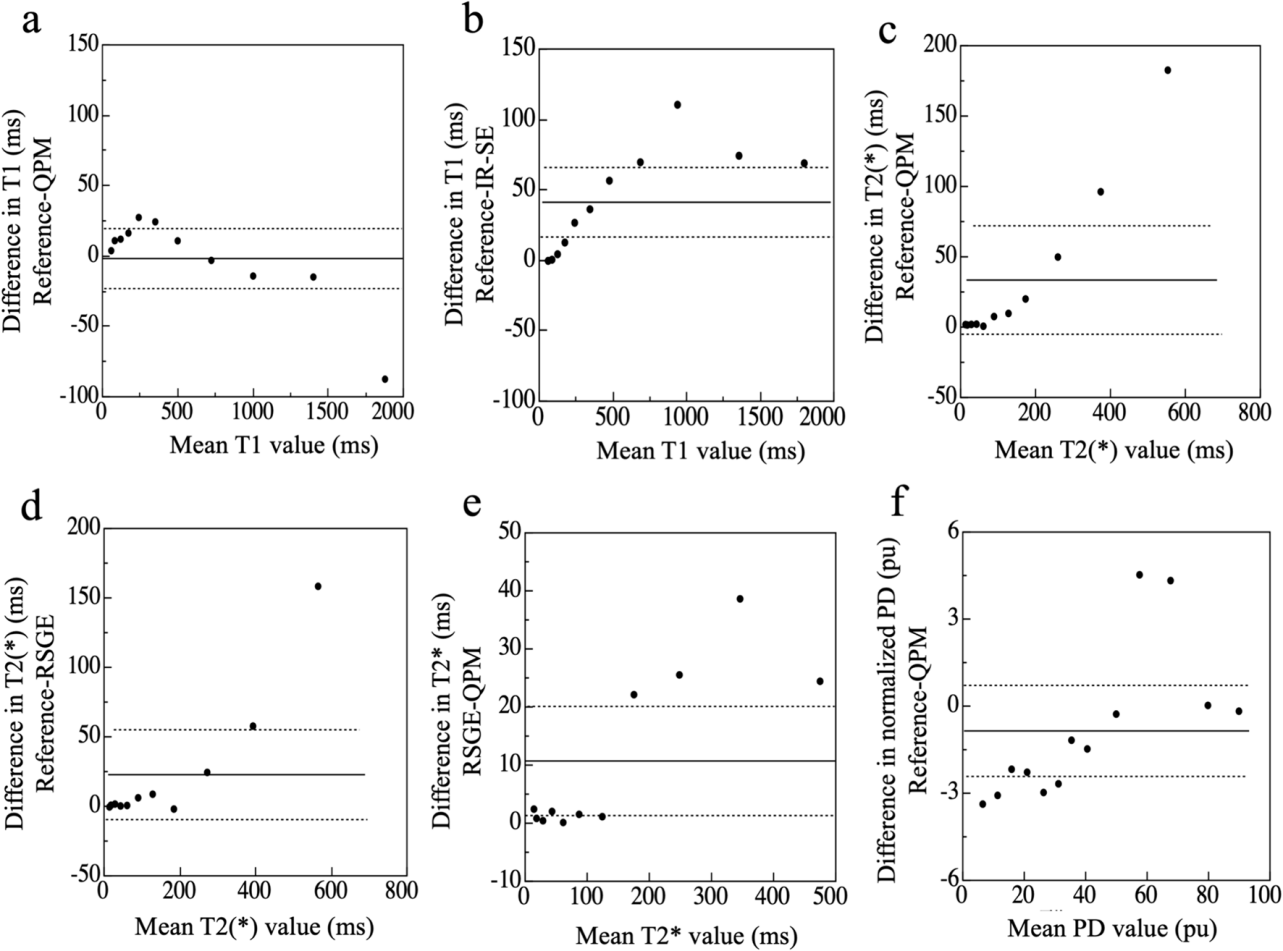
The Bland–Altman plots show the average *T*1 value of QPM (**a**) and IR-SE (**b**) for each *T*1 array compared with the reference value. The average bias of QPM was – 1.79 ms, and the 95% limits of agreement ranged from 19.53 ms to – 23.11 ms. The average bias of IR-SE was 41.2 6 ms, and the 95% limits of agreement ranged from 65.93 ms to 16.58 ms. Moreover, we show the average *T*2* (or *T*2) values of QPM (**c**) and RSGE (**d**) for each *T*2 array compared with the reference values. The average bias of QPM was 33.47 ms, and the 95% limits of agreement ranged from 72.04 ms to -5.10 ms. The average bias of RSGE was 22.76 ms, and the 95% limits of agreement ranged from 55.09 ms to – 9.56 ms. The comparison of RSGE and QPM showed an average bias of 10.71 ms, and the 95% limits of agreement ranged from 20.10 ms to 1.32 ms (**e**). Furthermore, we compare the average normalized PD values of each PD array obtained by QPM (**f**) with the reference values. The average bias of normalized PD value was – 0.85 pu and 95% limits of agreement ranged from 0.71 pu to – 2.42 pu

Figure 3 shows the Bland–Altman plots of *T*1/*T*2*/normalized PD values between the reference values and QPM/IR-SE/RSGE, and *T*2* values for RSGE and QPM. The average bias of *T*1 values was smaller for QPM (– 1.79 ms) compared to IR-SE (41.26 ms). The 95% limits of agreement for QPM and IR-SE ranged from 19.53 ms to – 23.11 ms, and from 65.93 ms to 16.58 ms, respectively. The trends of *T*2* values were similar for QPM and RSGE, as the average bias of *T*2* values was 33.47 ms and 22.76 ms for QPM and RSGE, respectively. The 95% limits of agreement for QPM and RSGE ranged from 72.04 ms to – 5.10 ms, and 55.09 ms to – 9.56 ms, respectively. The comparison of RSGE and QPM showed an average bias of 10.71 ms, and 95% limits of agreement ranged from 20.10 ms to 1.32 ms. The average bias of normalized PD value was – 0.85 pu and 95% limits of agreement ranged from 0.71 pu to – 2.42 pu.

**Fig. 4:**
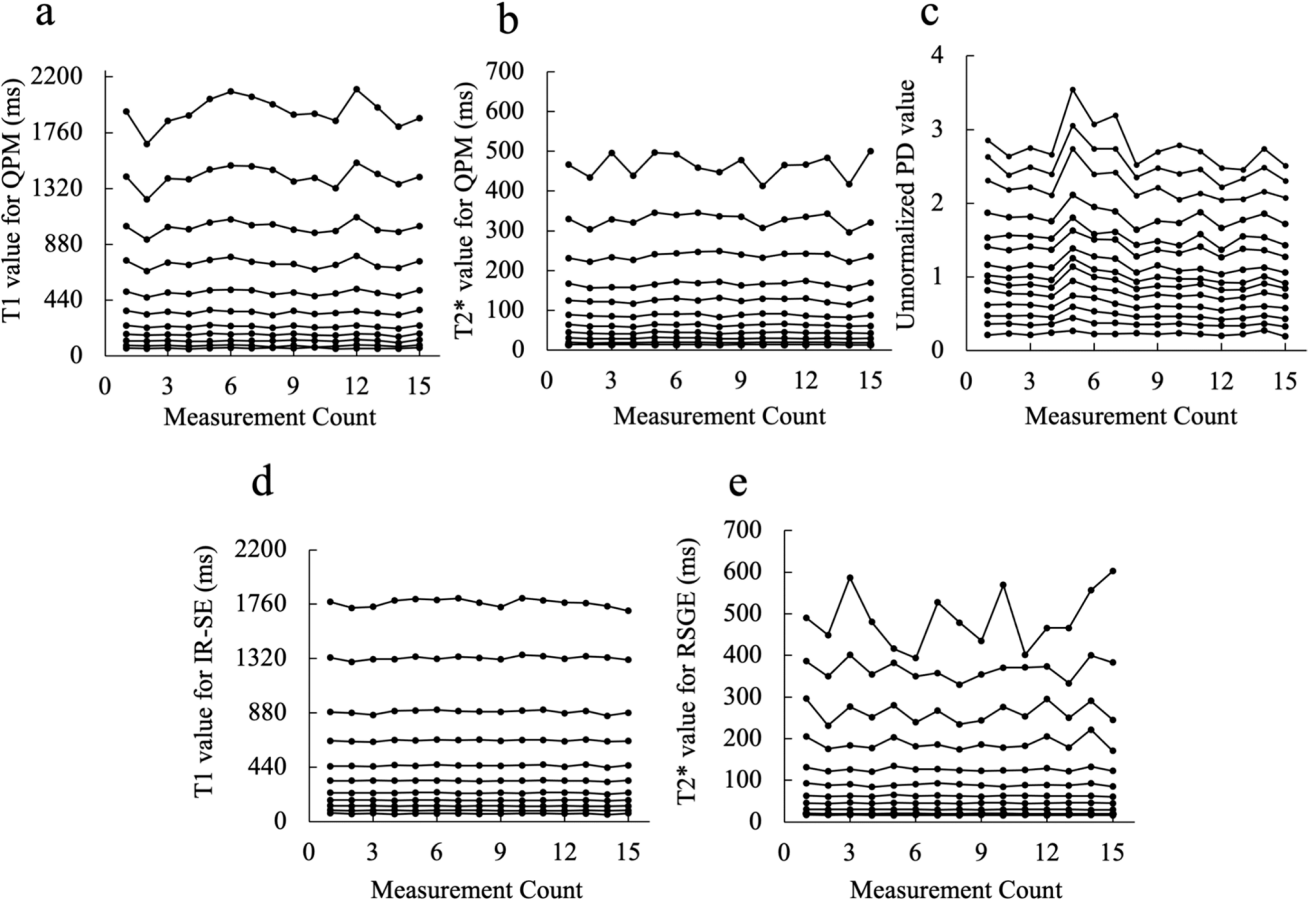
The daily *T*1 value (**a**), *T*2* value (**b**), and unnormalized PD value (**c**) obtained by QPM. Moreover, we show the daily *T*1 values obtained by IR-SE (**d**) and *T*2* values obtained by RSGE (**e**)

**Fig. 5:**
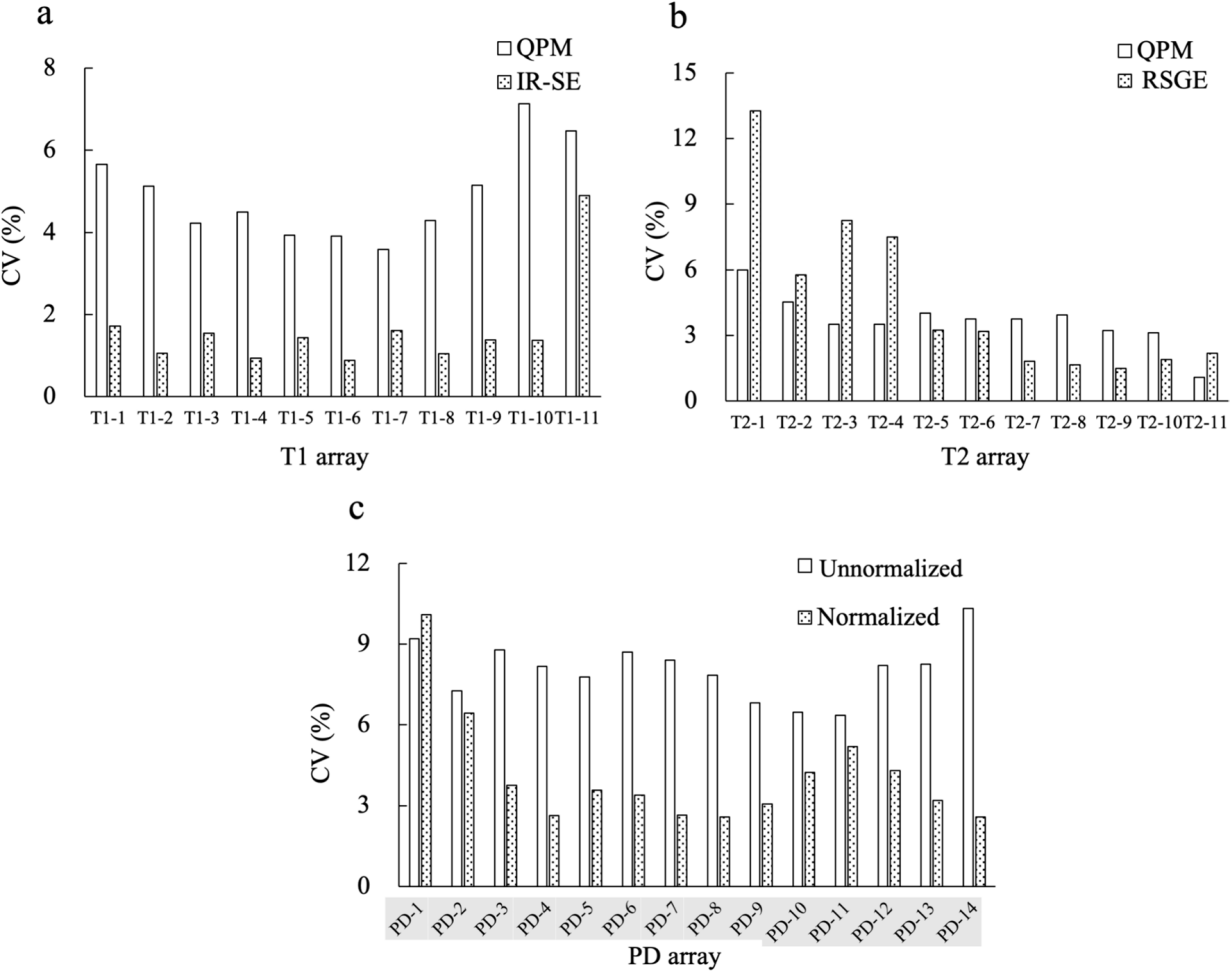
The coefficient of variations (CVs) of the *T*1 values measured by QPM and IR-SE (**a**). The CVs of the *T*1 values obtained by QPM and IR-SE of the entire *T*1 array were 4.91% and 1.62%, respectively. The CVs of the *T*2* value measured by QPM and RSGE (**b**). The CVs of the *T*2* values obtained by QPM and RSGE of the entire *T*2 array were 3.67% and 4.57%, respectively. The CVs of unnormalized and normalized PD values obtained by QPM (**c**). The CVs of the unnormalized and normalized PDs of whole PD array were 8.04 and 4.12%, respectively

Figure 4 shows the daily changes in the *T*1, *T*2*, and unnormalized PD values obtained by QPM, *T*1 values obtained by IR-SE, and *T*2* values obtained by RSGE. The CVs of the *T*1 values obtained by QPM and IR-SE with the entire *T*1 array were 4.91% and 1.62%, respectively (Fig. 5a). The CVs of the *T*2* values obtained by QPM and RSGE with the entire *T*2 array were 3.67% and 4.57%, respectively (Fig. 5b). The CVs of the unnormalized PD and normalized PD values obtained by QPM were 8.04 and 4.12%, respectively (Fig. 5c).

**Fig. 6:**
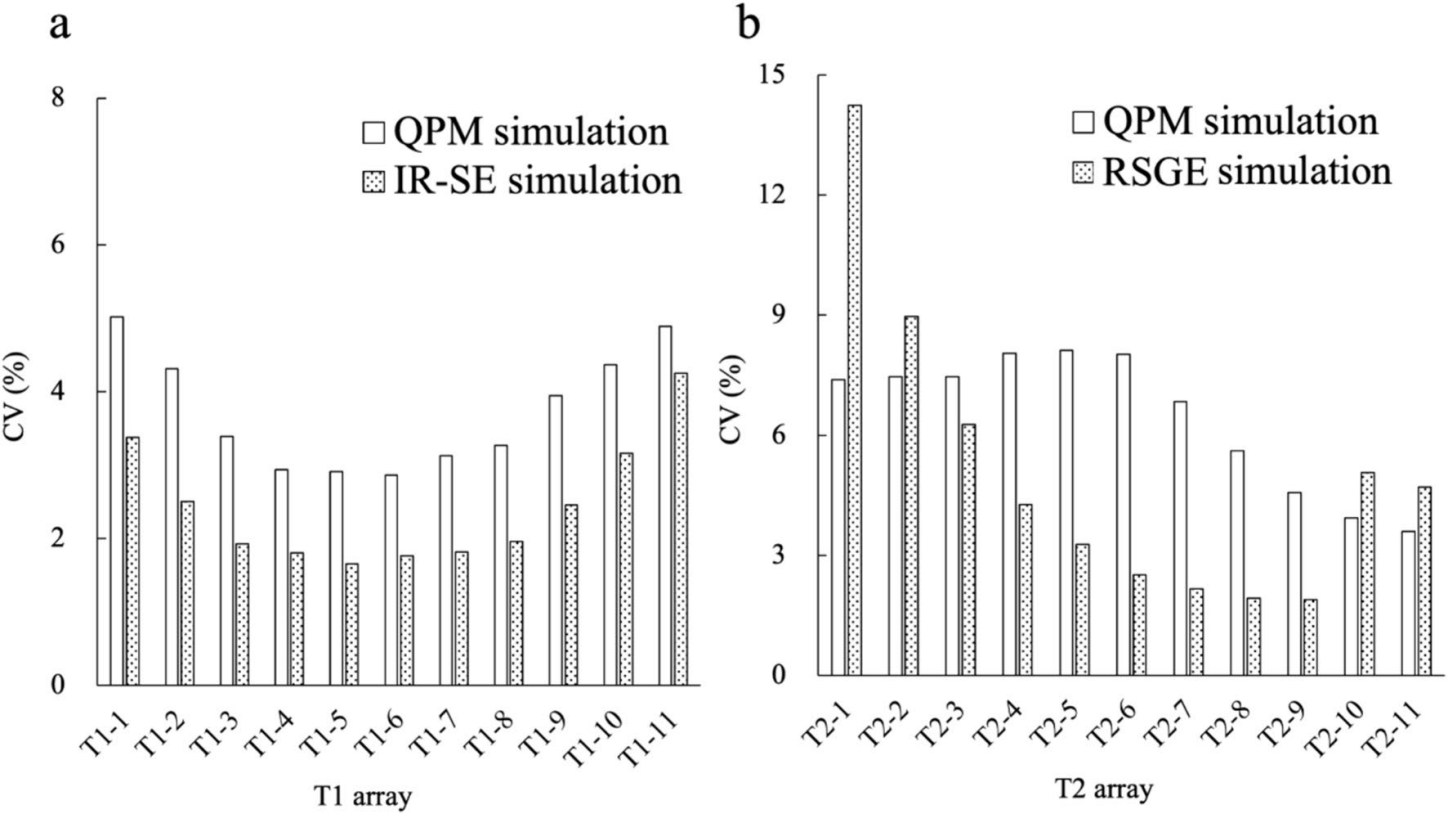
The results of the simulations of CVs obtained from the *T*1 array with QPM and IR-SE (**a**). The results of simulations of CVs obtained from the *T*2 array with QPM and RSGE (**b**)

Figure 6a shows the simulated CVs of QPM and IR-SE in the *T*1 arrays. The CVs of the QPM and IR-SE simulations are similar to those calculated from the scans. Figure 6b shows the CV simulation results for the QPM and RSGE in the *T*2 arrays. The CVs of the QPM and RSGE simulations are similar to those calculated from the scans. In the case of spheres with long *T*2 values (No. 1–4), the CV increased with decreasing *T*2* values in QPM, whereas the simulation CVs decreased.

## Discussion

In this study, we compared the relaxation times (*T*1, *T*2* value) and PD obtained through QPM with the reference values of the ISMRM/NIST MRI system phantom and conventional methods, which are considered the gold standard. The reference values of *T*2* for the ISMRM/NIST MRI system phantom have not been disclosed. Additionally, since *T*2* is dependent on voxel size, it was measured using RSGE in this study. The *T*1 and *T*2* values by QPM showed a strong correlation with the measured values by IR-SE/RSGE and the referenced values. Similarly, the unnormalized PD and normalized PD values obtained through QPM also demonstrated a strong correlation with the reference value. QPM showed fewer deviations from the Bland–Altman agreement limits compared to IR-SE. Additionally, QPM and RSGE exhibited similar trends. The CVs of *T*1, *T*2*, and normalized PD by the QPM were less than 5%.

However, the *T*1 values calculated from QPM showed an overestimation when compared to reference *T*1 values above 1000 ms and an underestimation for *T*1 values below 500 ms. These over- and underestimation of *T*1 values can be attributed to the magnetization transfer (MT) effect, FA uncertainty due to B1 + inhomogeneities, and scan parameters [13–15]. However, the effect of MT is small in in vitro studies such as this one, such as in phantoms, contrasting with in vivo effects with polymeric molecular structures [16]. The influence of B1 + inhomogeneities has been discussed by many researchers as part of the overestimation of T1 value measurement [14, 15]. Therefore, B1 + mapping techniques have been increasingly reported to improve MR scan availability at high fields and obtaining the correct RF profile using multisource parallel RF excitation [17]. QPM simultaneously calculated the quantitative values of *T*1, *T*2*, PD, and B1 using fitting formula (1) by setting the image intensity function formulated numerically obtained by Bloch simulation. Accordingly, we considered that the influence of B1 + inhomogeneities was theoretically small, because multiple quantitative values were calculated independently. Therefore, the *T*1 value measurement error in QPM may be due to the scan parameters set by the calculation. In this study, the QPM scan parameter set was optimized to minimize the fitting errors of the *T*1 and *T*2* values for the target scan tissue. However, the combination of *T*1 and *T*2* values assumed as brain tissues was separated from the reference values of the ISMRM/NIST MRI system phantom, except for WM(*T*2–7). Both the QPM *T*2* and RGSE *T*2* values linearly correlate with the reference *T*2 values, indicating that the influences that shorten *T*2 to *T*2* were consistently controlled throughout the experiments. The degree of QPM underestimation was particularly large for objects with long *T*1 and *T*2 reference values. One factor was that the longest QPM TE was 32.2 ms, which was shorter than those of the RSGE and reference values. The agreement between the QPM and RSGE measures from the Bland–Altman plots was high, and previous studies have shown similarly strong correlations [7]. We evaluated the accuracy of the PD measurements. PD values between different measurements are difficult to compare in absolute values, because the receiver sensitivity profile (RP) is adjusted for each measurement [18]. Therefore, the PD values have also been evaluated using the percentage unit (pu), which normalizes the PD of water as 100% [12]. In this study, the PD values of QPM were normalized using PD-14 (water: 100%) of the reference phantom based on this principle. Although the normalized PD values of QPM can achieve high accuracy, the normalization requires additional effort to set a reference region (ex. the ventricles for brain) in clinical scans. Such a reference region might be hard to find in some body scans. The necessitated additional effort or the lowered accuracy in measuring PD should be carefully considered in clinical application of QPM.

The repeatability of *T*1 and *T*2 values obtained with QPM was similar to that of the quantification technique for the ISMRM/NIST MRI system phantom (< 5%) [19, 20]. However, for objects with long *T*1 and *T*2 values, the CVs were more than 5%. This may be because of the effect of the eddy current. Jiang et al. discussed the possibility of underestimation by eddy currents in the case of *T*2 values longer than 500 ms [19]. Furthermore, the CVs of the QPM were smaller than those of the RSGE in spheres with longer *T*2 values (*T*2-1 and *T*2-2). In this study, the TE range of RSGE did not include a region of long *T*2 values in the ISMRM/NIST MRI system phantom. Because the range of the TE of RSGE was out of long *T*2 value spheres and the fitting was performed in a region of low *T*2* decay, the difference in the weak noise added to the signal might significantly affect the deviation of the *T*2* calculated value. The QPM CVs might be smaller than those of the RSGE in spheres with longer *T*2 values because the QPM is designed to avoid the accumulation of errors by rotating the phase at each echo. The CVs of unnormalized PD and normalized PD values obtained by QPM were 8.04 and 4.12%, respectively. This difference is due to the fact that unnormalized PD is influenced by the receiver sensitivity profile and other factors during long-term measurements, whereas normalized PD suppresses these influences. The results of this study are also consistent with reports indicating that neglecting RP inhomogeneities may result in errors of up to 8% [21]. Furthermore, the CVs for low normalized PD values (PD-1, 2) were more than 5%, but the CV decreased as the normalized PD values increased. This might be due to the increase in SNR caused by the larger PD [22].

The simulated CVs of QPM, IR-SE, and RSGE calculated for each sphere showed similar trends to those of the actual scans. This indicates that the possible influences (e.g., B1 intensity, spoiling accuracy, and liquid motion) other than noise are small. Examining each influence, the B1-intensity variation might not be so small because the unnormalized PD value of QPM shows considerable variance; however, the influence of the B1-intensity variation on the quantitative values is suppressed in the analysis of these methods. The variation in spoiling accuracy and the liquid motion might be small; however, the liquid motion might cause a difference between the simulation and actual *T*2* scans upon closer observation. For the spheres with long *T*2 (*T*2-1–*T*2-4), the CVs of QPM decreased as the *T*2* value decreased, whereas the CVs of the simulation were increased. The *T*2* values might have been underestimated because of the liquid convection motion caused by the vibration of the gradient coil. Previous studies have also reported underestimation at long *T*2 values [23, 24]. Similarly, in QPM, long *T*2* values were underestimated, and the denominator in the CV calculation formula was smaller, which might have increased the CVs.

Our study has several limitations. First, only the phantom was evaluated and no clinical images were used. QPM is a sequence that reduces the measurement error of the scan target by assuming the *T*1 and *T*2* values of human tissues. Therefore, it is essential to evaluate human tissues. However, there are bioethical limitations in evaluating human body using the gold standard IR-SE, since it requires a very long scan time. Thus, this study was simply an indispensable initial evaluation, using a phantom, to evaluate the correlation between QPM, the gold standard method, and the reference values, in addition to the reproducibility that is indispensable for quantitative techniques. Second, in this study, the used total scan time of about 42 min was longer than that used in actual clinical settings. This is because the acceleration factor of parallel imaging was minimized for obtaining a 23.1-cm-diameter spherical phantom without wraparound artifact in all directions and to minimize spatial noise inhomogeneity. The total scan time can be much shorter; for instance, it was reduced to about 10 min for a normal head size (average RL width 16 cm) by increasing the acceleration factor [10]. Although the estimated accuracy and repeatability may be decreased to roughly half in exchange for four times acceleration, caution should be exercised with spatially inhomogeneous decrease due to parallel imaging in detail. Third, QPM can be used to calculate multiple quantitative values, such as *T*1, *T*2 *, PD, B1, and QSM; however, the *T*1, *T*2*, and PD values were evaluated in this study. QSM has been reported to be clinically useful in many cases, and its clinical application is expected to expand in the future [25]. To the best of our knowledge, only QPM can be used to evaluate QSM in multiparametric sequences. It is necessary to evaluate the accuracy and reproducibility of the QSM obtained by the QPM in the future.

## Conclusions

The *T*1, *T*2*, and PD values obtained by QPM showed a strong correlation with both the measured and reference values, and repeatability was confirmed using the ISMRM/NIST MRI system phantom.

## Data Availability

The data that support the findings of this study are available from the corresponding author, [K. kudo], upon reasonable request.
